# Correction: The validity of mid-upper arm circumference as an indicator of underweight, overweight and obesity adults in Bangladesh

**DOI:** 10.1371/journal.pone.0335763

**Published:** 2025-10-29

**Authors:** Sheikh Arafat Rahman, Md. Humayan Kabir, Shaikh Shahinur Rahman, Md Kamruzzaman

The preposition “in” is missing from the title. The correct title is: The validity of mid-upper arm circumference as an indicator of underweight, overweight, and obesity in adults in Bangladesh. The correct citation is: Rahman SA, Kabir MH, Rahman SS, Kamruzzaman M (2025) The validity of mid-upper arm circumference as an indicator of underweight, overweight, and obesity in adults in Bangladesh. PLoS One 20(7): e0327499. https://doi.org/10.1371/journal.pone.0327499.
