# Supplementary figures and images for: The validity of mid-upper arm circumference as an indicator of underweight, overweight and obesity adults in Bangladesh
Source: PLoS One. 2025 Jul 28;20(7):e0327499. doi: 10.1371/journal.pone.0327499 (PMC12303288; doi:10.1371/journal.pone.0327499)

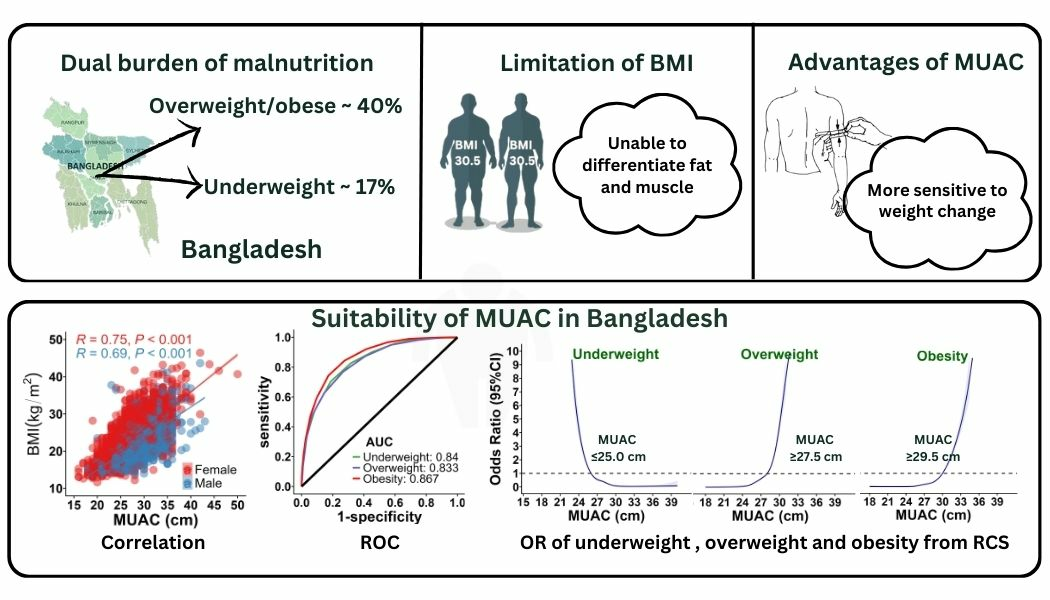

Supplement: S1 Fig — (TIF) [file pone.0327499.s001.tif]
