# Supplementary material for: The validity of mid-upper arm circumference as an indicator of underweight, overweight and obesity adults in Bangladesh
Source: PLoS One. 2025 Jul 28;20(7):e0327499. doi: 10.1371/journal.pone.0327499 (PMC12303288; doi:10.1371/journal.pone.0327499)
Supplement: S1 Table — (PDF) [file pone.0327499.s002.pdf]

Table S1: Multiple linear regression outcome of association between MUAC and BM

	MUAC (cm)		
	β	95%CI	P
BMI (kg/m^2)	0.81	0.74 to 0.88	**<0.001**
BMI^2 (kg/m^2)ᵵ	-0.01	-0.001 to -0.00	**<0.001**
Male	1.65	1.57 to 1.72	**<0.001**
Age (Years)	0.05	0.04 to 0.06	**<0.001**
Age^2 (Years)ᵵ	-0.001	-0.0006 to -0.00	**<0.001**

ᵵ: Quadratic term was used in the linear regression model
