# Supplementary material for: The validity of mid-upper arm circumference as an indicator of underweight, overweight and obesity adults in Bangladesh
Source: PLoS One. 2025 Jul 28;20(7):e0327499. doi: 10.1371/journal.pone.0327499 (PMC12303288; doi:10.1371/journal.pone.0327499)
Supplement: S2 Table — (PDF) [file pone.0327499.s003.pdf]

Table S2: Likelihood of underweight, overweight and obesity by MUAC quartile

		Underweight (BMI <18.5 Kg/m²)			***Overweight (BMI ≥23 Kg/m²)***			***Obese (BMI ≥27.5 Kg/m²)***		
	ŧMUAC Quartile	***Prevalence (%)***	***OR (95%CI)***	***P***	***Prevalence (%)***	***OR (95%CI)***	***P***	***Prevalence (%)***	***OR (95%CI)***	***P***
**Total†**	Quartile 1	***43.5***	***68.2 (48.4-96.3)***	***	7.5	1 (Ref)	--	*0.4*	1 (Ref)	--
	Quartile 2	13.2	13.5 (9.5-8.2)	***	28.5	4.9 (4.3-5.7)	***	3.0	8.9 (3.9-18.8)	***
	Quartile 3	6.0	5.6 (3.9-8.2)	***	49.0	12.4 (10.7-14.3)	***	8.3	22.1 (10.3-47.3)	***
	Quartile 4	1.1	1.0 (Ref)	--	**82.4**	***57.9 (49.6- 67.7)***	***	***34.7***	***143.3 (67.8-302.7***)	***
**Male**	Quartile 1	**54.3**	***65.6 (44.0-97.7)***	***	2.8	1 (Ref)	--	0.2	1 (Ref)	--
	Quartile 2	21.1	14.8 (9.9-22.0)	***	12.6	5.0 (3.4-7.5)	***	0.1	08 (0.1-5.5)	Ns
	Quartile 3	9.5	5.8 (3.8-8.8)	***	35.5	19.3 (13.3-27.9)	***	3.1	18.1 (4.4-74.8)	***
	Quartile 4	1.8	1 (Ref)	--	**71.9**	***89.6 (61.6-30.2)***	***	**20.4**	**142.6 (35.4-574.2)**	***
**Female**	Quartile 1	***38.5***	***136.5 (64.8-287.9)***	***	9.6	1 (Ref)	--	0.5	1 (Ref)	--
	Quartile 2	7.0	16.5 (7.7-35.4)	***	40.8	6.5 (5.5-7.64)	***	5.2	11.2 (6.1-20.4)	***
	Quartile 3	2.3	5.1 (2.3-11.5)	***	65.0	17.5 (14.7-20.7)	***	13.6	32.0 (17.8-57.5)	***
	Quartile 4	0.5	1 (Ref)	--	**92.8**	**121.43 (95.9-153.8)**	***	**49.0**	**194.6 (109.5-346.0)**	***
**Age 18-40 Years**	Quartile 1	***40.6***	***61.2 (39.5-95.0)***	***	7.6	1 (Ref)	--	0.4	1 (Ref)	--
	Quartile 2	13.2	13.6 (8.7-21.3)	***	27.3	4.6 (3.8-5.6)	***	3.0	8.6 (3.9-18.8)	***
	Quartile 3	5.3	5.0 (3.1-8.1)	**	48.8	11.7 (9.6-14.1)	***	7.3	22.1 (10.3-47.3)	***
	Quartile 4	1.1	1 (Ref)	--	**82.9**	***59.0 (48.0-72.5)***	***	**34.0**	***143.3 (67.8-302.7)***	***
**Age 40-60 Years**	Quartile 1	**42.8**	***69.4 (35.4-135.7)***	***	8.2	1 (Ref)	--	0.5	1 (Ref)	--
	Quartile 2	11.3	11.8 (5.9-23.6)	***	35.3	6.1 (4.6-8.1)	***	3.8	7.9 (2.8-22.6)	***
	Quartile 3	6.0	5.91 (2.9-12.1)	***	53.3	12.7 (9.6-16.9)	***	11.4	25.9 (9.5-70.8)	***
	Quartile 4	1.1	1 (Ref)	--	**84.2**	***59.4 (43.6-81.1)***	***	**37.6**	***121.9 (45.2-328.5)***	***
**Age ≥60 Years**	Quartile 1	**51.2**	***78.5 (29.0-212.5)***	***	6.3	1 (Ref)	--	0.4	1 (Ref)	--
	Quartile 2	16.4	14.64 (5.3-40.4)	***	22.2	4.2 (2.9-6.1)	***	1.7	4.5 (1.2-17.1)	***
	Quartile 3	9.1	7.5 (2.6-21.3)	***	48.2	13.8 (9.7-19.5)	***	6.6	18.1 (5.5-60.3)	***
	Quartile 4	1.3	1 (Ref)	--	**74.6**	***43.4 (29.4-63.9)***	***	**31.7**	***119.0 (37.3-379.5)***	***

ŧ: MUAC quartile 4 was considered as reference for underweight, while quartile 1 for overweight and obesity; †: Logistic regression model was adjusted for age, gender and area of residences; *: P<0.05; **: P<0.01; ***:P<0.001
