# Supplementary material for: The validity of mid-upper arm circumference as an indicator of underweight, overweight and obesity adults in Bangladesh
Source: PLoS One. 2025 Jul 28;20(7):e0327499. doi: 10.1371/journal.pone.0327499 (PMC12303288; doi:10.1371/journal.pone.0327499)
Supplement: S3 Table — (PDF) [file pone.0327499.s004.pdf]

Table S3: Morbidity (diabetes and hypertension) prevalence by proposed MUAC cut-off points by gender and age category

	Prediabetes % (95%CI)		Diabetes % (95%CI)		Prehypertension % (95%CI)		Hypertension % (95%CI)	
	MUAC	BMI	MUAC	BMI	MUAC	BMI	MUAC	BMI
Underweight	14.6 (13.5-15.8)	14.5 (13.0-16.1)	6.5 (5.7-7.4)	5.6 (4.7-6.7)	28.8 (27.4-30.2)	26.0 (24.2-27.9)	20.0 (18.8-21.3)	18.4 (16.9-20.1)
Normal weight	14.5 (13.3-15.8)	15.2 (14.2-16.2)	8.4 (7.4-9.4)	7.8 (7.1-8.5)	35.0 (33.4-36.7)	34.3 (33.1-35.6)	24.2 (22.825.7)	22.4 (21.3-23.6)
Overweight	17.2 (15.5-19.0)	17.1 (15.9-18.4)	11.8 (10.4-13.4)	12.3 (11.2-12.4)	36.9 (34.8-39.1)	34.9 (33.4-36.4)	31.5 (29.4-33.6)	35.5 (33.9-37.0)
Obesity	19.0 (17.7-20.3)	20.7 (18.6-23.0)	14.0 (12.9-15.3)	19.2 (17.1-21.5)	34.9 (33.4-36.5)	36.5 (34.0-39.1)	37.7 (36.1-39.3)	44.2 (41.6-46.9)

BMI: Body mass index; MUAC: Mid-upper arm circumference
