# Supplementary material for: The validity of mid-upper arm circumference as an indicator of underweight, overweight and obesity adults in Bangladesh
Source: PLoS One. 2025 Jul 28;20(7):e0327499. doi: 10.1371/journal.pone.0327499 (PMC12303288; doi:10.1371/journal.pone.0327499)
Supplement: Attachment — Submitted filename: Review PLOS ONE_22052025_rev.docx [file pone.0327499.s005.docx]

**Title:**
**The validity of mid-upper arm circumference as an indicator of underweight, overweight, and obesity among adults in Bangladesh**

This manuscript presents a well-conducted analysis using a nationally representative dataset (BDHS 2017–18) to propose sex- and age-specific mid-upper arm circumference (MUAC) cut-offs for identifying underweight, overweight, and obesity among adults in Bangladesh. The methods are statistically sound, and the manuscript is generally well-organized. However, several areas could be improved to strengthen the overall quality and clarity of the paper. As I do not have sufficient expertise in the advanced statistical methods applied in this study, I recommend that a statistician review the analytical components to ensure their appropriateness and robustness.

**Introduction:**

1. The introduction is well-written, but could be made more concise by removing some repetitive information.
2. While the strengths of MUAC are discussed, its limitations are not adequately addressed. For example, MUAC measurements may be less accurate in individuals with conditions like edema or muscle hypertrophy or variations in measurement technique. In some circumstances, it might be best to complement MUAC with other anthropometric indicators to provide a more complete picture.

**Methods:**

1. Please justify the selection of age groups (18–40, 40–60, >60). Were these based on previous studies, expert opinion, or data-driven criteria?
2. Line 222: The Youden Index is mentioned as the method for determining cut-off points. Please ensure consistent spelling throughout the text.
3. Lines 227–230: Restricted cubic spline analysis is appropriate, but the number and placement of knots should be explained, e.g., was it based on the AIC criteria?.
4. Line 237: Please use decimal points (.) consistently in accordance with English language conventions, rather than commas (,).
5. Please indicate whether any data were missing and how missing values were handled.
6. Consider moving the ethical approval and data access information into a separate subsection titled “Ethics Approval and Data Availability” to improve clarity and organization.

**Results:**

1. The Results section is quite dense and could be difficult to follow. Adding subheadings for different analysis components could enhance readability.
2. Please define all abbreviations in tables and figures, including “+PV” and “–PV,” in the footnotes to ensure they are self-explanatory.

**Discussion:**

1. The discussion does not explain why MUAC cut-offs are lower in older adults or the biological basis of sex-specific differences. Please consider discussing age-related muscle mass loss and fat redistribution as possible explanations.
2. Some odds ratios reported (e.g., OR > 100) appear extremely large. It would be helpful to caution readers about potential overestimation due to stratification or sparse data.
3. Consider elaborating on how these MUAC cut-offs could be used in clinical and community settings and whether further research is needed to validate their use.
4. The discussion could be expanded to include examples of how MUAC could be applied in the field, such as for community health worker (CHW) screening or integration into national nutrition surveillance systems.
5. Several relevant limitations are not mentioned, including:
   - MUAC measurements can be distorted by conditions such as edema or muscle hypertrophy.
   - No discussion is provided on measurement reliability (e.g., inter- or intra-observer variability), which is important for field-based anthropometric tools like MUAC.

Conclusions:

1. The conclusion notes the use of MUAC but could be expanded by adding some suggestions for how it could be integrated into health systems or programs.
2. Authors could add a sentence about whether there is a need for **validation with body composition measures** and **implementation studies**.
