# Supplementary material for: The validity of mid-upper arm circumference as an indicator of underweight, overweight and obesity adults in Bangladesh
Source: PLoS One. 2025 Jul 28;20(7):e0327499. doi: 10.1371/journal.pone.0327499 (PMC12303288; doi:10.1371/journal.pone.0327499)
Supplement: Attachment — Submitted filename: Response to Reviewers.docx [file pone.0327499.s007.docx]

June 05, 2025

To

The Editor

PLOS One

**Subject:** Response to the reviewer’s comments for the manuscript entitled “*The validity of mid-upper arm circumference as an indicator of underweight, overweight and obesity in adults*”.

**Ref: Manuscript ID: PONE-D-25-18917**

Dear Editor,

Thank you for your email and for reconsidering our submission. We have addressed the issues raised by the editor and reviewer. A rebuttal letter, which provides our responses, is appended.

We have attached tracked and clean version of the manuscript.

We hope that the revised manuscript will prove acceptable for publication.

Kind Regards,

Md. Kamruzzaman

Dept. of Applied Nutrition and Food Technology

Islamic University, Kushtia, Bangladesh

Email: [md.kamruzzaman@anft.iu.ac.bd](mailto:md.kamruzzaman@anft.iu.ac.bd), mkzaman.m@gmail.com

**Response to the reviewer’s comments**

**Reviewer 1:**

**Introduction:**

1. The introduction is well-written but could be made more concise by removing some repetitive information.

***Response:*** *Repetitive information has been removed from introduction and corrected.*

(Page 4)

2. While the strengths of MUAC are discussed, its limitations are not adequately addressed. For example, MUAC measurements may be less accurate in individuals with conditions like edema or muscle hypertrophy or variations in measurement technique. In some circumstances, it might be best to complement MUAC with other anthropometric indicators to provide a more complete picture.

***Response:*** *Thanks for this comment. Limitations of MUAC has been added in the introduction section.*

(Page 5, Line 120)

**Methods:**

1. Please justify the selection of age groups (18–40, 40–60, >60). Were these based on previous studies, expert opinion, or data-driven criteria?

***Response:*** *We thank the reviewers for this observation. The chosen age groups (18–40, 40–60, >60 years) were determined based on a combination of physiological relevance and alignment with existing literature concerning age-related variations in body composition and nutritional risk.*

(Page 8, Line 215)

2. Line 222: The Youden Index is mentioned as the method for determining cut-off points. Please ensure consistent spelling throughout the text.

***Response:*** *Thanks for this comment. Youden index has been corrected consistently.*

(Page 9 Line 240)

3. Lines 227–230: Restricted cubic spline analysis is appropriate, but the number and placement of knots should be explained, e.g., was it based on the AIC criteria?

***Response****: Thanks for this important comment. The number of knots and model fit has now added the methodology section.*

(Page 10, Line 244)

4. Line 237: Please use decimal points (.) consistently in accordance with English language conventions, rather than commas (,).

***Response:*** *Thanks for this comment. Decimal point has been corrected now.*

(Page 10, Line 256)

5. Please indicate whether any data were missing and how missing values were handled.

***Response:*** *Missing data are included in the methodology section.*

(Page 7, Line 179)

6. Consider moving the ethical approval and data access information into a separate subsection titled “Ethics Approval and Data Availability” to improve clarity and organization.

***Response:*** *Thanks for this comment. Ethics Approval and Data Availability section has been separated.*

(Page 7, Line 185)

**Results:**

1. The Results section is quite dense and could be difficult to follow. Adding subheadings for different analysis components could enhance readability.

***Response:*** *Thanks for this comment. We have now divided the results into different subsections.*

2. Please define all abbreviations in tables and figures, including “+PV” and “–PV,” in the footnotes to ensure they are self-explanatory.

***Response:*** *All abbreviations including +PV and -PV has been abbreviated.*

(Page 13, Line 309)

**Discussion:**

1. The discussion does not explain why MUAC cut-offs are lower in older adults or the biological basis of sex-specific differences. Please consider discussing age-related muscle mass loss and fat redistribution as possible explanations.

***Response:*** *Thanks for this comment. Causes of lower MUAC cut-offs points in older people has now been discussed in the discussion section.*

(Page 18, Line 422)

2. Some odds ratios reported (e.g., OR > 100) appear extremely large. It would be helpful to caution readers about potential overestimation due to stratification or sparse data.

***Response:*** *We appreciate the reviewer's helpful comment regarding the very large odds ratios (OR >100) found in some subgroup analyses. We agree that these might show sparse data or a few events, which can lead to unstable or exaggerated estimates. This has now been acknowledged as a limitation of data sparse bias.*

(Page 19 Line 454)

3. Consider elaborating on how these MUAC cut-offs could be used in clinical and community settings and whether further research is needed to validate their use.

***Response:*** *We appreciate this valuable suggestion. We have expanded the practical implications of our MUAC cut-offs, highlighting their potential usefulness in both clinical and community health environments. In addition, we recognize the necessity for further validation studies to verify the generalizability and prognostic value of these thresholds. This information has been included in the discussion section.*

(Page 18, Line 437)

4. The discussion could be expanded to include examples of how MUAC could be applied in the field, such as for community health worker (CHW) screening or integration into national nutrition surveillance systems.

***Response:*** *We appreciate the reviewer's helpful suggestion. We have added more details to the discussion section. Now, we include examples of how MUAC can be used in real-life situations.*

(Page 18, Line 437)

5. Several relevant limitations are not mentioned, including:
   - MUAC measurements can be distorted by conditions such as edema or muscle hypertrophy.
   - No discussion is provided on measurement reliability (e.g., inter- or intra-observer variability), which is important for field-based anthropometric tools like MUAC.

***Response:***

- *We appreciate this comment. Effect of edema or muscly hypertrophy on MUAC measurement has been acknowledged in the limitations section.*

*(Page 19, Line 459)*

- *Thanks for tis comment. The data, that we have used, are secondary in nature and in the data definition, there are no information on inter or intra observation variability. Therefore, we are unable discuss on measurement reliability. However, we have acknowledged that in the limitation section.*

*(Page 19, Line 462)*

Conclusions:

1. The conclusion notes the use of MUAC but could be expanded by adding some suggestions for how it could be integrated into health systems or programs.

***Response:*** *We appreciate this comment. Use of MUAC has been expanded in the conclusion section.*

*(Page 20, Line 479)*

2. Authors could add a sentence about whether there is a need for **validation with body composition measures** and **implementation studies**.

***Response:*** *We appreciate this comment. Need for validation with body composition measures and implementation studies has already been added in the discussion section.*

*(Page 19, Line 466)*

**Reviewer 2:**

1. I suggest to add to abstract that the cut-off point were selected by Younden index.

***Response:*** *Cut-oof based on Youden index has been added now.*

*(Page 2, Line 52)*

2. I suggest to summarize it to avoid repetition.

***Response:*** *Introduction has been edited to avoid repetition.*

3. I suggest to add the word adults throughout the work.

***Response:*** *Word adults has been added throughout the work.*

4. I failed to see if you include or exclude pregnant women.

***Response:*** *Thanks for this comment. Inclusion of non-pregnant has been added in the methodology section.*

*(Page 7, Line 180)*
